# Isolation and characterization of lytic bacteriophages from sewage at an egyptian tertiary care hospital against methicillin-resistant *Staphylococcus aureus* clinical isolates

**DOI:** 10.1016/j.sjbs.2022.03.019

**Published:** 2022-03-19

**Authors:** Safia Samir, Amira El-Far, Hend Okasha, Rania Mahdy, Fatima Samir, Sami Nasr

**Affiliations:** a Biochemistry and Molecular Biology Department, Theodor Bilharz Research Institute (TBRI), Giza, Egypt; b Microbiology Department, Theodor Bilharz Research Institute (TBRI), Giza, Egypt

**Keywords:** MRSA, Lytic bacteriophages, Sewage, Transmission electron microscopy, SDS-polyacrylamide gel electrophoresis, Polymerase chain reaction, Restriction digestion, *Caudovirales*, Mitomycin C, TBRI, Theodor Bilharz research Institute, *S. aureus*, *Staphylococcus aureus*, PCR, Polymerase chain reaction, CLSI, clinical and laboratory standards institute, OX, Oxacillin, FOX, Cefoxitin, ESKAPE, (*Enterococcus faecium, Staphylococcus aureus, Klebsiella pneumoniae, Acinetobacter baumannii, Pseudomonas aeruginosa,* and *Enterobacter species*), MRSA, Methicillin Resistant *Staphylococcus aureus*, LB, Luria-Bertani, MitC, mitomycin C, PFU, Plaque forming unit, AMR, antimicrobial resistance, MDR, Multidrug-resistant, ITS, Internal transcribed spacer, NGS, double-stranded, ds, next generation sequencing, PTA, phosphotungstic acid, TEM, Transmission electron microscopy

## Abstract

**Background:**

Methicillin resistant *Staphylococcus aureus* (MRSA) is a pathogen to humans causing life-threatening infections. MRSA have the capability to grow resistance to many antibiotics, and phage therapy is one treatment option for this infection.

**Objectives:**

The aim of the present study was to isolate and characterize the lytic bacteriophages specific to MRSA from domestic sewage water at a tertiary care hospital in Egypt.

**Methods:**

Thirty MRSA strains were isolated from different clinical samples admitted to the microbiology lab at Theodor Bilharz Research institute (TBRI) hospital, Giza, Egypt. They were confirmed to be MRSA through phenotypic detection and conventional PCR for *mecA* gene. They were used for the isolation of phages from sewage water of TBRI hospital. Plaque assay was applied to purify and quantify the titer of the isolated phages. The host range of the isolated phages was detected using the spot test assay. The morphology of phages was confirmed using transmission electron microscope (TEM). Digestion of DNA extracted from phages with endonuclease enzymes including *EcoRI* and *SmaI* was performed. SDS-PAGE was performed to analyze MRSA specific phage proteins. As a positive control prophages were isolated from a mitomycin C (MitC) treated culture of *S*. *aureus* strain ATCC25923. Further characterization using conventional polymerase chain reaction (PCR) was used to select three known Staphylophages by detecting the endolysin gene of phage K, the polymerase gene of phage 44AHJD, and the minor tail gene of phage P68.

**Results:**

Isolated phages in this research displayed a wide host range against MRSA using the spot test, out of thirty tested MRSA isolates 24 were sensitive and got lysed (80%). The titer of the phages was estimated to be 1.04 × 10^6^ pfu/ml using plaque test. Identification of head and tail morphology of the phages was achieved using TEM and they were designated to tailed phages of order *Caudovirales*, they composed an icosahedral capsid. Prophages were isolated through MitC induction. DNA of phages was digested by endonuclease enzymes. Conventional PCR yielded 341 bp of phage K endolysin gene and phage P68 minor tail protein gene 501 bp. Protein analysis using SDS-PAGE showed 4 proteins of sizes between 42 kDa and 140 kDa.

**Conclusion:**

Phages isolated here are alike to others mentioned in previous studies. The high broad host range of the isolated phages is promising to control MRSA and can be in the future commercially suitable for treatment as lysate preparations. Animal models of phage-bacterial interaction will be our next step that may help in resolving the multidrug resistant crisis of MRSA in Egypt.

## 1 Introduction

Around the world, antibiotic resistance is an escalating crisis that presents big health and economic load (Targets, 2021). By 2050, the World Health Organization has estimated that if there are no effective alternatives found to current antibiotics, antimicrobial resistance (AMR) may signify a global cost of USD 100 trillion and up to 10 million deaths yearly, surpassing the numbers of cancer and heart diseases (Neill JO’. , 2014). Phage therapy is an underexplored alternative to antibiotic treatment, bacteriophages are used to infect and kill pathogenic multidrug-resistant (MDR) bacteria (Lu and Koeris, 2011). The mechanism and dynamics of bacterial lysis make phage therapy a radically different treatment to antibiotics due to phage naturally self-amplify, producing phage progeny during infection that binds and lyses target cells. Phages are specific to their targets and produce lesser endotoxin after cell lysis, in addition to that they are more capable to break through biofilms. Thus, the probability of phages application to cause adverse side effects like disruption of the gut microbiome is less. On the contrary to that, broad-spectrum antibiotics may cause the death of normal flora (Samir, 2021).

It is a challenging task to search for a suitable and effective phage against a range of similar but different strains of pathogenic bacteria (Nagel et al., 2016). Many phages may be unfit for use in phage therapy despite their likeable targeting characteristics, this is simply because they are lysogenic phages and not lytic. Usually, temperate phages or prophages are ineligible from phage therapy due to that their infection cycle may unpredictably result in either lysis of host or integration into its genome. They may encode virulence factors such as toxins, they can protect their host from infection by other phages, and their lifecycle predisposes them to be agents of horizontal gene transfer (Hyman, 2019).

Globally, the *Enterococcus faecium, Staphylococcus aureus, Klebsiella pneumoniae, Acinetobacter baumannii, Pseudomonas aeruginosa,* and *Enterobacter species* (ESKAPE) pathogens lead to healthcare-associated infections. *Staphylococcus aureus* is one of them (Vale de Macedo et al., 2021, El-Far et al., 2021). MRSA is responsible for a variety of diseases ranging from soft tissue and skin infections to life-threatening situations such as pneumonia, bacteremia, and sepsis (Boswihi and Udo, 2018). Using of phages for MRSA treatment can be locally for local infections, intraperitoneal administration for systemic infe ctions, and orally. Endolysins (or lysins) are produced by bacteriophages and have been effectively used to control antibiotic-resistant pathogenic bacteria found on mucosal surfaces and in infected tissues with a low chance of bacterial resistance (Rahimzadeh et al., 2017).

Several phages from the order *Caudovirales* are obligatorily lytic on *S. aureus* and cannot transfer bacterial DNA. Most of them have a complex virion structure comprising a head and long contractile tail and have been classified to the family *Herelleviridae* (Barylski et al., 2020). Some, less abundant and comprising only 14 isolates of sequenced genomes have a short tail. They follow the *Picovirinae* subfamily of family *Podoviridae*. Regardless of being found in different topographical areas, phages that infect *S. aureus* are much alike (Gozdek et al., 2018), and have been interrelated to the *Rosenblumvirus* genus, subfamily *Picovirinae* (previously 44AHJD like phages or 68-like phages) (Glowacka-Rutkowska et al., 2019).

Phage identification and purification to be free of bacterial cells, toxins and other compounds are essential to be an adequate therapeutic option for patients suffering from resistant bacterial infection (Rios et al., 2016). Negative staining of purified viruses and electron microscopy remains the gold standard in the identification of bacteriophages. Polymerase chain reaction (PCR) is a simple method to confirm the subsistence of phages faster than plaque assays, based on the detection of nucleic acid. Also, it is likely to nominate different phage lineages in a few short hours, with unnecessity for whole-genome sequencing (Ács et al., 2020). Using lytic phages as biocontrol factors signifies the demand for the most appropriate and standard methods to ensure application safety (François et al., 2016). Sewage water is recognized to be a rich source of bacteriophages. Hospital sewage represents a very selective environment where resistant bacteria are found (Mattila et al., 2015). Previous studies reported the identification of phages specific to several bacteria including *Escherichia coli*; *Pseudomonas aeruginosa*; *Acinetobacter* sp., *Klebsiella* sp., and *Enterococcus faecalis* from wastewater. Phages specific to MRSA have not been extracted from wastewater in Egypt (Kwiatek et al., 2012, Shen et al., 2012). Therefore, the present study aimed to recognize the lytic bacteriophages specific to pathogenic SA/MRSA clinical isolates at a tertiary care hospital in Egypt from indigenous sewage water sources using different microbiological and molecular techniques.

## 2 Materials and methods

### 2.1 Bacterial strains collection and culture conditions

Thirty clinical isolates were collected from different specimens such as urine, blood, and wounds of patients at Theodor Bilharz Research Institute hospital. All isolates were cultured on mannitol salt agar medium (Oxoid, England) and the plates were incubated for 24 h at 37 °C. Identification of *S. aureus* was done using Gram staining and biochemical tests such as catalase, coagulase, and DNase (HiMedia, India). *S. aureus* ATCC 25,923 was used as a laboratory reference bacterium for molecular and microbiological assays. Bacterial isolates were stored at −70 °C in the form of glycerol stocks until needed for further processing (Sambrook and Russell, 2001).

### 2.2 Genotypic detection of S. aureus

The *S. aureus* ATCC 25,923 and the collected bacterial isolates were subjected to conventional PCR assay for detection of *16srRNA* gene using specific primers. A commercially available DNA extraction kit (QiaAmp DNA Mini kit) was used for genomic DNA extraction according to the manufacturer’s instructions. The primer pairs used are *16srRNA* forward 5′- ACGGTCTTGCTGTCACTTATA-3′, reverse 5′-TACACATATGTTCTTCCCTAATAA-3′ (Johnson et al., 2016). The thermal profile was as follows: initial denaturation was done at 95 °C for 10 min, the samples were subjected to 30 cycles of denaturation at 95 °C for 1 min, 50 °C for 1 min and at 72 °C for 1 min. A general extension at 72 °C for 10 min. PCR evaluated by electrophoresis on 2% agarose gel containing ethidium bromide (0.2mg/ml) in the presence of a 100-bp ladder (Fermentas) as a DNA molecular weight marker. positive results for *16srRNA* will give a band of 257bp Johnson et al., 2016.

### 2.3 Phenotypic detection of MRSA

According to the clinical and laboratory standards institute (CLSI) (Weinstein et al., 2020), phenotypic detection was performed using the disk diffusion method. As recommended oxacillin (OX) and cefoxitin (FOX) were applied on Muller-Hinton agar (Oxoid, England) plates containing 4% NaCl. Isolates characterized with a zone of inhibition less than 21 mm for oxallicin and 13 mm for cefoxitin are MRSA.

### 2.4 Genotypic detection of MRSA

Isolates of MRSA-DNA phenotype were extracted and examined by PCR for detection of *mecA* gene that resist oxacillin. The *mecA* gene was amplified by specific primers. The primers used are *mecA* forward 5′-GTAGAAATGACTGAACGTCCGATAA-3′, reverse 5′- CCAATTCCACATTGTTTCGGTCTAA-3′ (Askar et al., 2016). The thermal profile was as follows: initial denaturation was done at 95 °C for 10 min, the samples were subjected to 30 cycles of denaturation at 95 °C for 1 min, annealing at 47 °C for 30 sec and extension at 72 °C for 30 sec. And general extention at 72 °C for 10 min. PCR products were recorded by electrophoresis on 2% agarose gel containing ethidium bromide (0.2 mg/ml), in the presence of a 100-bp ladder (Fermentas) as a DNA molecular weight marker. Positive results for *mecA* gene will produce a band of 310 bp (El-Far et al., 2021).

### 2.5 Wastewater (sewage) Sample Collection for Bacteriophage Isolation

The bacteriophages specific to *S. aureus* (MRSA) were isolated from several wastewater samples that were taken from sewage water of TBRI hospital. Samples were collected in capped 50 ml falcon.

#### 2.5.1 Sewage sample processing for bacteriophage isolation

Twenty ml of raw sewage water was mixed with 20 ml phage buffer (2 mM CaCl_2_, 10 mM MgSO_4_, 50 mM Tris base pH = 7.6, and 150 mM NaCl), and shaken at 225 rpm overnight at 4 °C to dislodge phages from their counterpart bacterial hosts. The mixture was then centrifuged for 20 min/4000 rpm to separate bacterial cells and debris, working in biological safety cabinet (under sterile conditions) supernatant was filtered through 0.45 µm and 0.2 µm sterile filters. About 400 µl chloroform was added (1% per volume) to get rid of the remaining bacterial remnants and then incubated for 15 min/37 °C. Finally, the sample was stored in a dark bottle at 4 °C till needed for work (Abatángelo et al., 2017).

#### 2.5.2 Bacterial sample culturing and bacteriophage enrichment

Under aseptic conditions, twenty µl of bacterial glycerol stocks (MRSA clinical strain and standard strain of *S. aureus* ATCC 25923) were inoculated into 2 ml Luria-Bertani (LB) broth (10 g tryptone, 10 g NaCl, and 5 g yeast extract per liter) and incubated overnight at 37 °C. Twenty µl of overnight cultures were inoculated into 2 ml LB broth and incubated for 3 hrs/37 °C (to reach exponential phase culture). Two ml of the 3hrs bacterial culture was mixed with 2 ml of sewage filtrate and 5 ml LB broth and incubated overnight at 37 °C with shaking at 80 rpm. The culture was centrifuged for 20 min/4000 rpm; supernatant lysate was filtered through 0.2 µm Millipore sterile filter. This enrichment step for phage amplification was repeated three times. The phage filtrate (supernatant lysate) was stored in a dark bottle at 4 °C till needed for work to be examined for lytic phages using spot assay and plaque assay (Hallajzadeh et al., 2020). For long-term storage, 0.5 ml of phage lysate was mixed with 0.5 ml sterile glycerol to obtain a final concentration of 50%, and stored at −70 °C.

### 2.6 Spectrophotometric optical density measurement of phage treated liquid culture

Phage filtrate was added to an equal volume of a broth culture of bacteria and incubated at 37 °C. Cell lysis was monitored at time intervals of 2hrs, 6hrs, and 24 hrs. This was indicated by the loss of bacterial culture turbidity. OD_600_ was measured for both bacterial sample and infected bacterial sample with phage using SE6100 UV– VIS double beam spectrophotometer (Abbot) (Jensen et al., 2015).

### 2.7 Induction of prophages using Mitomycin C (MitC)

To induce phage using MitC in *S. aureus* strain ATCC 25923. Overnight culture in LB broth was diluted 1:100 and allowed to grow for 2 hrs at 37 °C on the shaker incubator (190 rpm) till reaching OD_600_ 0.2 before MitC was added to a final concentration of 0.5 μg/ml (50 μl of 2 mg/ml MitC stock to 20 ml culture). The same diluted culture without MitC was used as a control. Incubation proceeded for 24hrs and the turbidity at 600 nm was monitored at 1 hr intervals. One ml from 1 hr induced culture was centrifuged at 4000 rpm/20 min/4°C. The bacteria were pelleted to the bottom of the tube, while the supernatant was filtered through 0.2 µm filter and collected into 1 ml eppendorf tube and stored at 4 °C refrigerator till processed. The bacterial pellet was resuspended in LB broth and left for growth at 37 °C for 3 hrs. In an LB (10g tryptone,10g NaCl, 5g yeast extract, and 15g/L agar) agar plate, 10 µl of 3hrs bacterial culture was mixed with 3 ml top agar (4 g/L agar, 4mM MgCl₂, and 4 mM CaCl₂) and poured onto the plate till solidification. Spots of ten µl from the filtrate of induced 1hr culture were spotted onto the plates of MitC induced *S. aureus* ATCC 25923 and other tested MRSA strains and then incubated upright overnight at 37 °C. Next day, clearing zones of phages were observed in the agar indicating lysis (Protocol MCI).

### 2.8 Assessment of enriched filtrate efficacy by spot testing and host range analysis

To evaluate the efficacy of the enriched filtrate obtained from sewage water against other MRSA isolates collected in the microbiology lab. Under aseptic conditions, thirty MRSA isolates were cultured in LB broth overnight at 37 °C and then sub-cultured by addition of 5 ml LB broth to 50 µl from overnight culture and grown at 37 °C for 3 hrs. One hundred µl of 3 hrs culture was inoculated into 3 ml of molten top agar overlaid onto LB agar plates. Each overlay was allowed to solidify for 30 min. Spots of 10 µl of the enriched phage lysate were spotted onto the MRSA isolates overlay, dried, and then incubated at 37 °C overnight. Results were analyzed visually based on the detection of any lysis (Jensen et al., 2015).

### 2.9 Plaque assay (agar overlay method) and determination of bacteriophage titer

Under aseptic conditions, one MRSA isolate was processed for plaque production using the enriched phage lysate extracted from it. The MRSA isolate was cultured in LB broth overnight at 37 °C and then sub-cultured by addition of 5 ml LB broth to 50 µl from overnight culture and grown at 37 °C for 3 hrs (to reach exponential phase culture). Phage suspension was 10-fold serially diluted in phage buffer (from 10^−1^ till 10^−6^). One hundred  µl of 3hrs MRSA culture was added to 100 µl of each phage dilution, incubated 10 min/ 37 °C, and then mixed with 3 ml of molten LB top agar. This was poured quickly onto the surface of a solidified LB agar plate. Plates were incubated overnight at 37 °C. Plaques presence was checked visually and counted. Phage titer was calculated using the formula:

PFU/ml = PFU counted/ dilution × volume of phage dilution plated (Nasser et al., 2019).

(PFU: plaque-forming unit).

### 2.10 Transmission electron microscopy (TEM)

Fresh phage samples were processed for TEM in 2 ways: 1st way: spots or plaques less than 48 h old were picked up and suspended in 50 µl CaCl_2_ then sent to the TEM lab. 2nd way: one ml of sterile high-titer lysate (10^8^ phages/ml) was centrifuged at 13000 rpm/1hr/4°C. The supernatant was discarded and the pellet was re-suspended in cold 0.1 M of ammonium acetate solution pH = 7 (sterile filtered). The mixture was centrifuged again 13000 rpm/1hr/4°C and the procedure was repeated another time. Nine hundred and fifty µl of the supernatant was aspirated leaving behind 50 µl of solution and left overnight at 4 °C to allow the pelleted phage to diffuse into the liquid (Giuseppe Aprea et al., 2015). For image preparation, 5 µl of the specimen was spotted onto a carbon-coated 400-mesh copper acid grid for 3–5 min and stained with 2% of phosphotungstic acid (PTA) stain (pH = 7) for 30–60 s. It was examined by Zeiss transmission electron microscope (Carl Zeiss LEO EM 906 E, Germany) at an accelerating voltage of 100 kV in the central lab, Cairo University, CURP, Research Park, Giza, Egypt. Morphology and phage dimensions (head diameter, head length, and tail length) were detected and the results were compared to a known dimension of staphylophages (such as phage K).

### 2.11 Phage DNA preparation and amplification

#### 2.11.1 Phage DNA preparation

Spots of bacteriophages were scratched using a tip then DNA was extracted using a commercially available DNA extraction kit (QiaAmp DNA Mini kit) for genomic DNA extraction according to the manufacturer’s instructions. The purity and concentration of the DNA obtained were determined through 260/280 nm absorbance measures using the NanoDrop spectrophotometer 2000c (Thermo Scientific) (García-Alegría et al., 2020). Additionally, extraction of phage nucleic acid was also achieved from phage lysate. Ten ml of phage lysate produced from enrichment on MRSA bacterial culture was mixed with 5 ml of 10% PEG 8000 and 1 M concentration of NaCl and incubated overnight at 4 °C. The mix was centrifuged at 10,000 rpm for 15 min. The pellet was re-suspended in 500 µl of SM buffer (100mMNaCl, 10mMMgSO4, 10 mMTris-HCl [pH = 7.5]) and transferred to a 2ml eppendorf tube. Five µl of 12.5 mg/mL RNase A was added to the tube and incubated for 30 min at 37 °C. Twelve µL of 20% SDS and 5 µl of 10 mg/ml proteinase K were added to the mixture and incubated for 30 min at 37 °C. The remaining extraction steps were carried out according to Sambrook *et al.,* (Sambrook and Russell, 2001). Ten µl of phage DNA was resolved by electrophoresis on 0.7% agarose gel with DNA molecular weight marker (Estrella et al., 2016).

#### 2.11.2 PCR of selected phages

Using NCBI primer designing tool – NIH (https://www.ncbi.nlm.nih.gov/tools/primer-blast/), the genome sequence of phage K is deposited in the GenBank database under accession number AY176327.1. The genome of 44AHJD (GenBank accession no. AF513032) and the genome of P68 (GenBank accession no. AF513033). The following primers were designed: the endolysin gene was amplified from phage K endolysin forward 5′- AAGTTACCGCCTACTGGAGC-3′, reverse 5′- GTACCAACTGCCTGTCCTCA-3′. The polymerase gene was amplified from phage 44AHJD polymerase forward 5′- TGGCATACCTGCATTACGTTC-3′, reverse 5′- ATCGGGTCGAATAAACTGGGG-3′. The minor tail protein gene was amplified from phage P68 minor tail protein forward 5′- GCTGGGTATCCACCATTGCTA-3′, reverse 5′- TGCTGGTAAACTTGAAGCGT-3′. The PCR cycling program consisted of 95 °C for 3 min, then 30 cycles of 1 min at 95 °C, 1 min at 58 °C, and 1 min at 72 °C, with an additional step of 5 min at 72 °C. PCR products were analyzed by electrophoresis on a 1.5% agarose gel containing ethidium bromide (0.2 mg/ml).

### 2.12 Restriction analysis of phage DNA

The extracted nucleic acid was digested with restriction endonucleases: *EcoR*I and *Sma*I (Thermo Scientific, USA). The fragmented DNA was separated using 10% PAGE stained with ethidium bromide (Tan et al., 2020).

1. Preparation of protein extracts of bacteriophages and SDS-PAGE technique

A 10 ml of phage suspension was treated with 96% ethanol and incubated for 24 hrs at −20 °C. Following removal of ethanol, the precipitated protein was resuspended in 2x loading buffer to analyze by 10% SDS-polyacrylamide gel resolving gel (Tris-HCl buffer with pH 8.8) and 6% polyacrylamide in Tris-HCl buffer with pH6.8 was used as stacking gel. Electrophoresis was carried out using Tris-glycine chamber buffer at 100 mA constant current. A molecular weight standard (Thermo Scientific™ Spectra™ Multicolor Broad Range Protein Ladder 10 to 260 kDa, Catalog number: 26634) was used in the electrophoretic separation, after that the gel was stained with Coomassie Brilliant blue G 250 (Sigma-Aldrich C.I. 42655).

### 2.13 Statistical analysis

The data were presented as the mean ± SD. The test of significance was performed by GraphPad Prism 8 (San Diego, California, USA) using multiple T-test. The *p*-value less than 0.05 was considered to indicate a statistically significant difference.

## 3 Results

### 3.1 Bacterial isolates

Thirty *S.aureus* isolates were collected from the microbiology lab at Theodor Bilharz Research Institute from various clinical samples, including blood (15/30, 50%), urine (7/30, 23.4%), ascitic fluid (4/30, 13.3%), and pus (4/30, 13.3%). The percentage of isolates recovered from males and females was 73.3% (22/30) and 26.7% (8/30), respectively. The highest proportions of isolates were from patients 45 years and older, with a rate of 70% (21/30).

### 3.2 Genotypic detection of S. Aureus

**Fig. 1:**
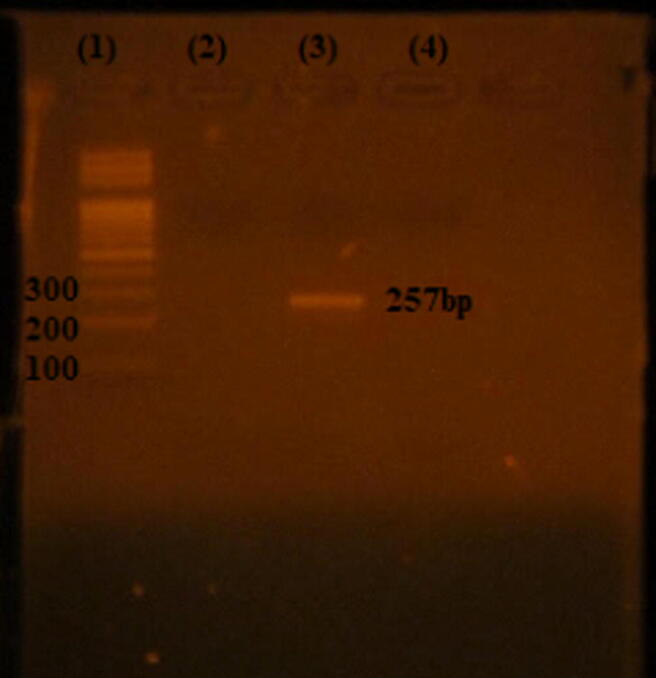
PCR product of *16srRNA* of *S. aureus*. Lane 1: 100–1000 bp DNA ladder (Fermentas), Lane 3: 257 bp PCR product Lane 4: negative control.

The 257 bp gene product for *16srRNA* (Fig. 1) was found in 100% of isolates.

### 3.3 Phenotypic and Genotypic detection of MRSA

**Fig. 2:**
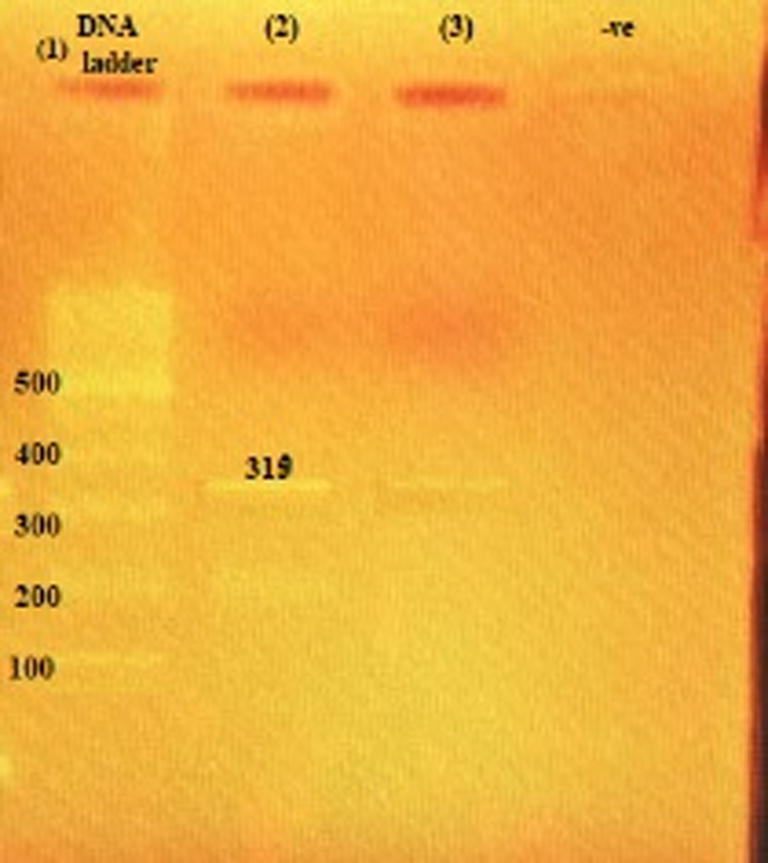
Agarose gel electrophoresis of amplified product *mec A* gene generated by PCR. Lane 1: 100 bp molecular weight marker (Fermentas); Lanes (2–3): positive samples showing 310 bp of the methicillin-resistant *mecA* gene; -ve: negative control.

MRSA isolates were detected phenotypically and genotypically. Conventional PCR for detection of *mecA* gene showed that all the isolates carried the *mec A* gene. The 310 bp gene products are shown in Fig. 2.

### 3.4 Spectrophotometric optical density measurement of phage treated liquid culture

**Fig. 3:**
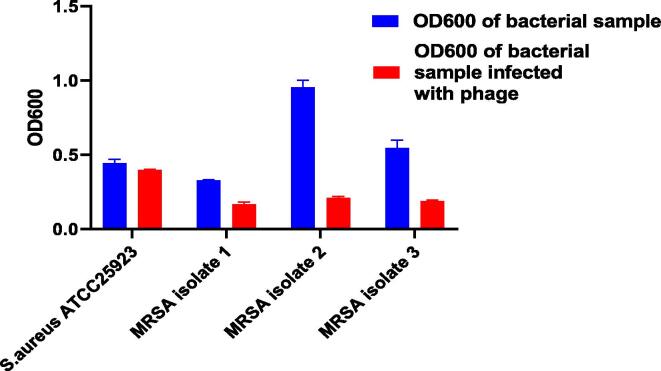
This graph represents the difference of OD_600_ readings between normal bacterial cultures and bacterial cultures infected with phage.

OD_600_ readings of *S.aureus* ATCC 25,923 reference strain & 3 MRSA isolates were measured three times and the mean with SD was calculated as shown in Table 1 & Fig. 3.

**Table 1:** OD_600_ records of *S.aureus* (reference strain & 3 MRSA isolates).

Bacteria	Reading of bacterial sample OD_600_	Reading of bacterial sample infected with phage OD_600_	p-value
*S.aureus* ATCC 25,923	0.44 ± 0.025	0.396 ± 0.006	0.035
MRSA isolate 1	0.327 ± 0.006	0.167 ± 0.015	0.00007
MRSA isolate 2	0.95 ± 0.047	0.21 ± 0.01	0.00001
MRSA isolate 3	0.546 ± 0.051	0.188 ± 0.007	0.0003

**Fig. 4:**
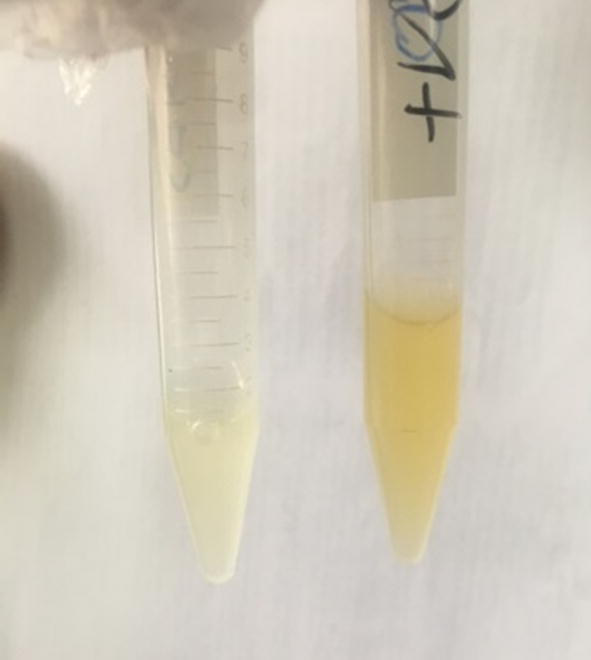
Bacterial growth retarded by phage treatment for 24hrs (left tube), the tube on the right is a positive sample containing bacterial culture only.

The results show that the reading of OD_600_ *S.aureus* ATCC 25,923 was nearly the same as that of a bacterial sample infected with phage (turbidity was nearly equal by naked eye). While the MRSA isolate OD_600_ reading decreased when infected with phage indicating that it has a lytic effect on that isolate and bacterial growth was retarded compared with that of the MRSA culture without infection (weak turbidity was noticed indicating weak bacterial growth) Fig. 4. The enriched phages obtained after processing and enrichment against the 3 MRSA isolates were assessed against the remaining 27 MRSA isolates collected in the microbiology lab.

### 3.5 Prophages induction with MitC

Monitoring of MitC induced phage showed a successful release in 1hr of *S. aureus* culture. Induction by MitC was accompanied by cell lysis, as indicated by the decrease in the optical density at 600 nm.

### 3.6 Spot testing and host range assay

**Fig. 5:**
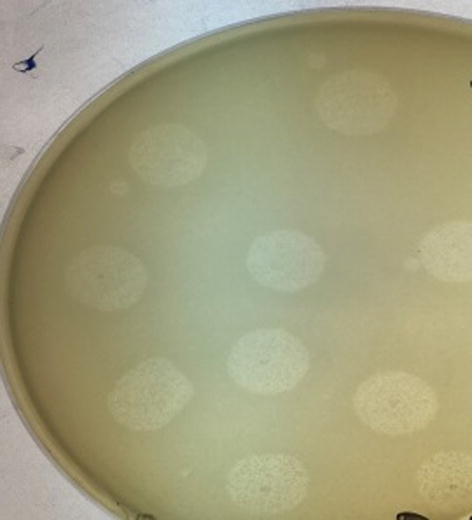
spot assay.

The major aim of this study is the isolation of phages specific to SA/MRSA with a broad host range. Spot testing on the collected isolates indicated that bacteriophages found in enriched filtrates of sewage were able to lyse 24 out of 30 MRSA isolates (80%). Zones of lysis in the spot test are shown in Fig. 5.

### 3.7 Plaque assay and titer determination

**Fig. 6:**
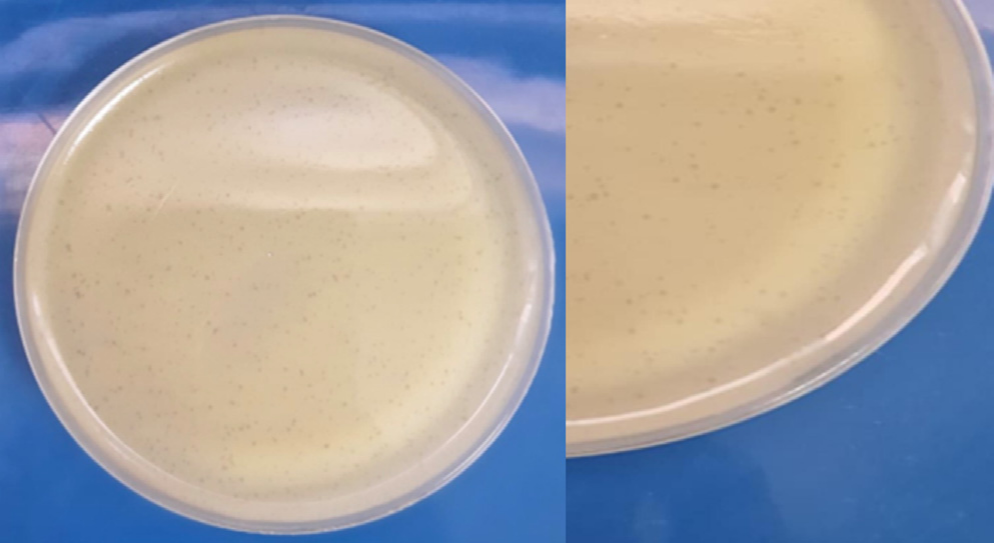
small-sized plaques of Staphylophages.

Small-sized plaques were obtained using the agar overlay method. The plaques were clear zones of lysis. The phage titer was ascertained by serial dilution. Plaques were counted after overnight incubation at 37 °C and the titer of the isolated phage was determined to be 1.04 × 10^6^ pfu/ml. Fig. 6 shows plaques of the bacteriophages.

$$\begin{array}{c} \mathrm{Phage}\;titer\;was\;calculated\;for\;dilution10^{-3}\\ Pfu/ml=pfu\;counted/dilution\times volume\;of\;phage\;dilution\;plated\\ =104/10^{-3}\times 100\;\mu l \end{array}$$

= 1.04 × 10^6^ pfu/ml, which is the volume of original solution (stock) that actually got plated in plate with dilution 10^−3^.

### 3.8 TEM and morphology of phages

**Fig. 7:**
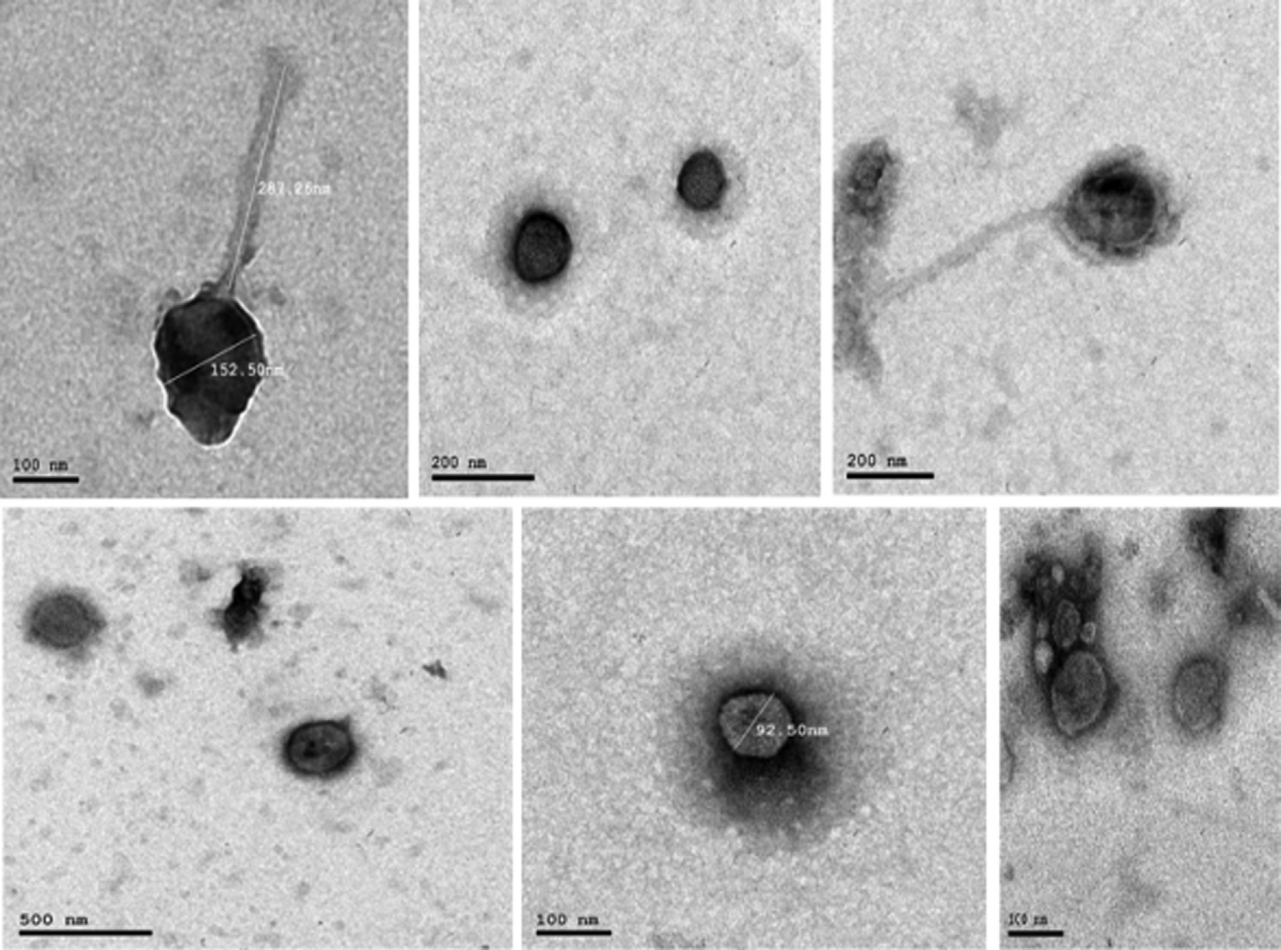
morphology of MRSA bacteriophages examined by TEM. The images of phages are with icosahedral heads, long non-contractile tails, and very short non-contractile tails.

TEM micrographs obtained from high-titer lysates showed that the virion morphology of the phages was similar to order *Caudovirales* which are tailed bacteriophages with an icosahedral head that contains the viral genome and is attached to the tail by a connector protein (Turner et al., 2021) as shown in Fig. 7.

### 3.9 Phage DNA extraction & PCR for selected phages

**Fig. 8:**
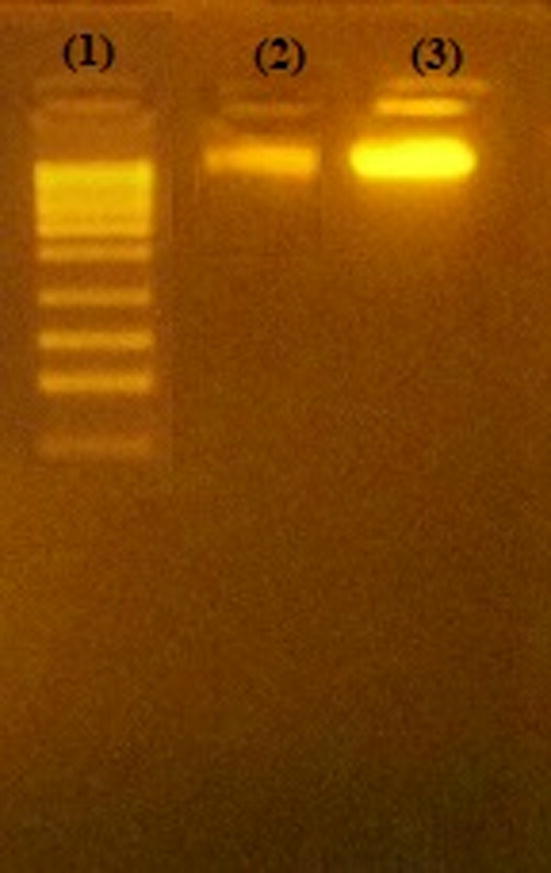
Electrophoresis bands of phage DNA. Lane (1): DNA ladder XXL (250 bp- 25 kbp) ready-to-use (GeneOn). Lane (2): DNA of prophage induced from *S. aureus* using MitC. Lane (3): DNA of phages isolated from sewage.

**Fig. 9:**
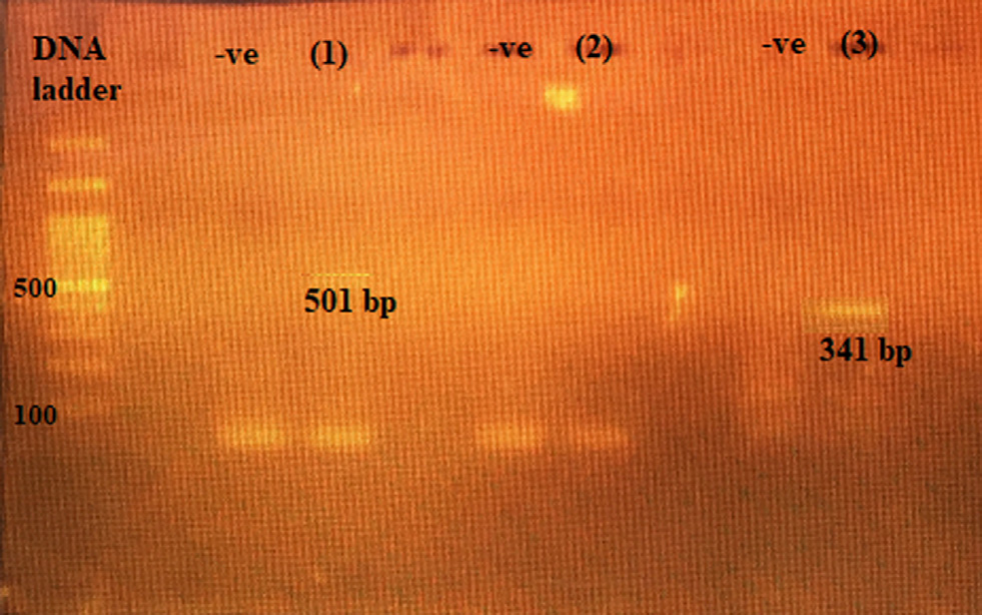
Agarose gel electrophoresis of amplified product phage p68 minor tail protein and phage K endolysin gene generated by PCR. Lane DNA ladder: (ladder 100 bp); Lane (1): 501 bp of P68 minor tail protein gene; -ve: negative control, lane (2): No band for 44AHJD polymerase gene; -ve: negative control, lane (3): 341 bp of phage K endolysin gene; -ve: negative control.

Extracted DNA from phages isolated from sewage and phages isolated from MitC induction are shown in Fig. 8. Conventional PCR on DNA extracted from sewage for detection of phage K endolysin gene showed a band of 341 bp, and phage P68 minor tail protein gene 501 bp. However, phage 44AHJD polymerase gene 270 bp was not detected. Fig. 9.

### 3.10 Restriction analysis of the phage genome

According to NEB cutter web tool (https://nc2.neb.com/NEBcutter2), the genomes of three phages (phage K, phage P68, and phage 44AHJD) were analyzed. We chose two endonuclease enzymes (*EcoR*I and *Sma*I) to differentiate between the three phages. Data shown in Table 2 represented the restriction sites of each enzyme.

**Table 2:** Restriction sites of restriction enzymes for phage K, phage P68, and phage 44AHJD.

	Phage K MW 148317 bp			Phage P68 MW 18227 bp			Phage 44AHJD MW 16,748		
	No. of cuts	Cut Position	Expected bands MW	No. of cuts	Cut Position	Expected bands MW	No. of cuts	Cut Position	Expected bands MW
*EcoR*I	1	2	148315 bp 2 bp	1	12,471	12471 bp 5756 bp	1	11,034	11034 bp 5714 bp
*Sma*I	1	679	147638 bp 679 bp	non	Non	non	Non	Non	non

**Fig. 10:**
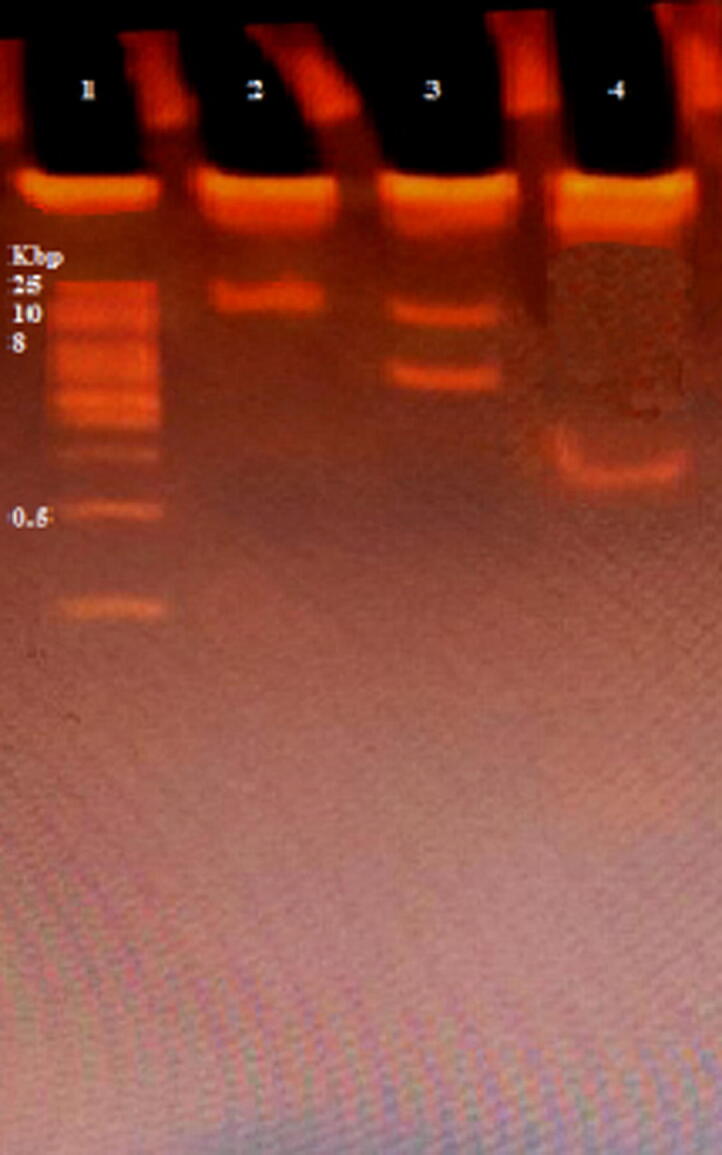
Restriction batterns analysis of phage genome DNA using two enzymes. Lane (1): DNA Ladder XXL (250 bp − 25 kbp), ready-to-use (GeneOn). Lane (2): undigested phage. Lane (3): Digestion using *EcoR*I. Lane (4): Digestion using *Sam*I.

The results of digestion provided in Fig. 10 indicated the presence of phage K and phage P68. Digestion with *EcoR*I yielded 2 bands. Nevertheless, digestion with *Sma*I yielded a band at MW higher than 500 bp.

### 3.11 Phage protein analysis

**Fig. 11:**
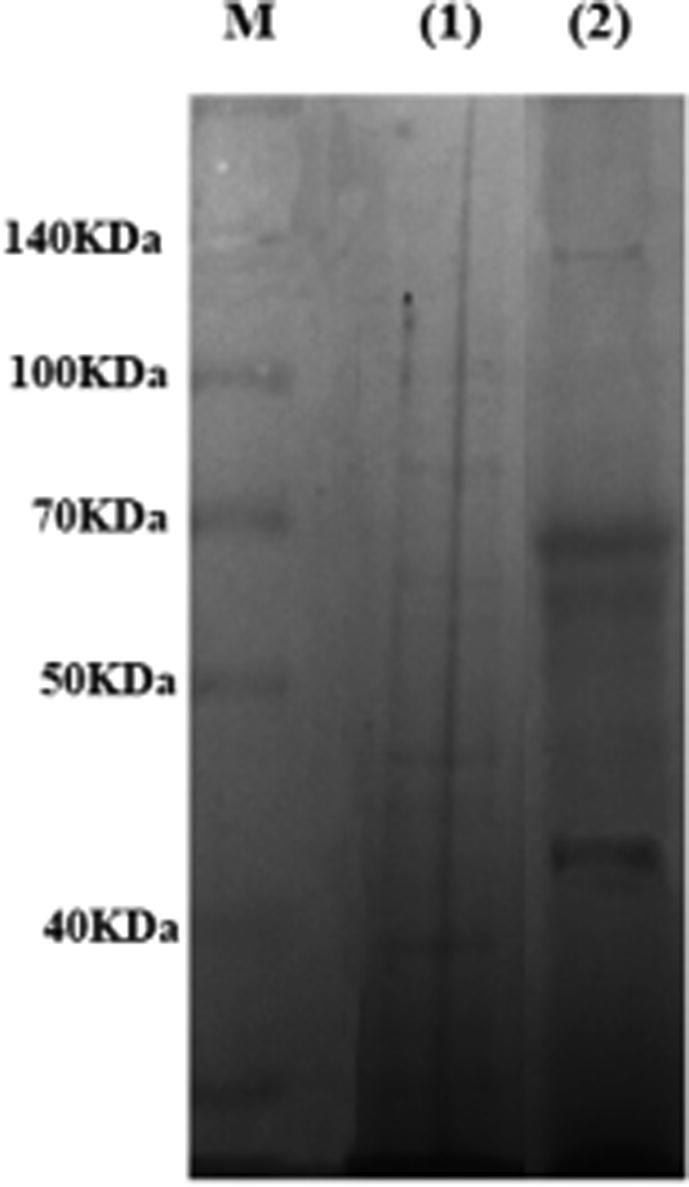
SDS-PAGE analysis of proteins isolated from MRSA-specific phage. Lane M: Protein Ladder10 to 260 kDa (Thermo Scientific™ Spectra™ Multicolor Broad Range), lane (1): MRSA proteins, and lane (2): phage proteins.

Phages’ proteins were isolated on 10% SDS-PAGE. Phages’ proteins shown in Fig. 11 clarified 4 protein bands approximately 140, 68, 65 kDa in weight, and 42 kDa compared to sizes of bands of the known molecular weight protein standard.

**Fig. 12:**
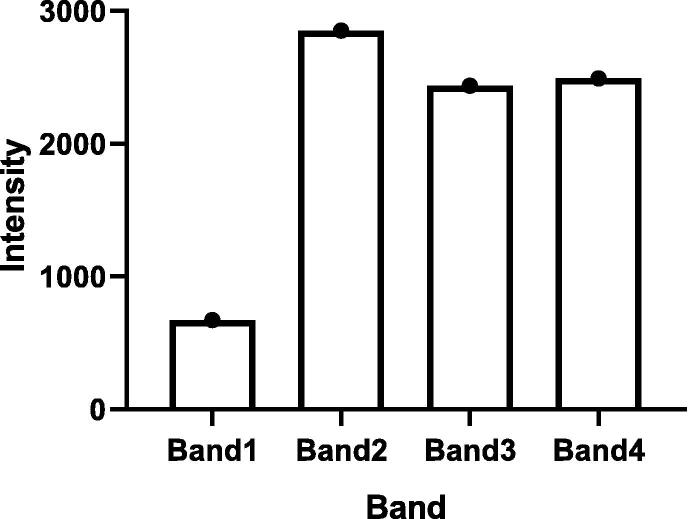
This graph represents the intensity of each band in SDS-PAGE of phage protein.

Results of analysis of SDS-PAGE using GelAnalyzer software are shown in Table 3 and Fig. 12. The results obtained confirmed the presence of 4 bands with high intensity at varied MW.

**Table 3:** Comparison of band intensity of SDS-PAGE using GelAnalyzer.

Bands of phage from high MW to low MW	RF	Intensity
1.	0.313	762
2.	0.4	2852
3.	0.716	2437
4.	0.971	2490

Where RF is defined as the migration distance of the protein through the gel divided by the migration distance of the dye front.

## 4 Discussion

Failure of treatment of SA/MRSA infections by routinely used antibiotics is a rising crisis in Egypt (Tadesse et al., 2017). Little information is known about the bacteriophages that are present in Egypt against our local Staphylococcal and MRSA strains. In this research, we aimed to highlight the phage therapy role as a probable solution and as an alternate to resolve the drug-resistant antibiotic crisis of MRSA. Moreover, our purpose was to extract and recognize the phages using reliable techniques (Bonilla et al., 2016;2016.).

A total of 30 *S.aureus* (MRSA) isolates were identified phenotypically and genotypically at the microbiology lab at Theodor Bilharz Research Institute from various clinical samples including blood, urine, pus, and ascitic fluid. A study documented a high prevalence of *S. aureus* in blood and surgical wounds samples (Gupta, 2011). Moreover, another study showed that the recurrence of MRSA in patients having burns was 24% (Naqvi and Hashmi, 2007). The next important step was to examine sewage samples extracted from different sources of TBRI on these isolates to isolate phages specific for SA/MRSA.

Biogeographic study of bacteriophages has proposed that abundance of bacteriophages may not be equal at all locations and that factors including source choice, temperature, amount of disinfectant used in sewage, the flow rate of sewage, exposure to sun or radiation are important and affect phages’ amount in the starting material of extraction (Tan et al., 2020, Abedon et al., 2017). Our study showed that the isolation of phages specific to MRSA isolates from sewage water taken from different locations in the hospital was forthright. The explanation of the richness of our wastewater with phages is that they are enriched with bacteria from the hospital setting, and that make available a tremendous host range for all types of phages (Rasool et al., 2016). Wang et al., (Wang et al., 2016) in China stated that staphylophages were found in large amounts in fecal sewage. Another study from Malaysia showed the easiness of bacteriophage isolation from sewage (Tan et al., 2020). On the contrary, a study done by Mattila et al., (Mattila et al., 2015) in Finland showed that it was not easy to isolate phages against MRSA from sewage drains. The efficacious recovery of bacteriophages capable of killing precise pathogenic bacteria is dependent on host density and surrounding environmental conditions (Echeverría-Vega et al., 2019).

The isolated phages in this study displayed lytic activity against SA/MRSA, and the host range of these phages was determined using spot testing that showed clear spots of lysis drawn by infection of phages to the susceptible bacterial host. Additionally, this lytic activity was observed in phage treated bacterial culture through the measurement of optical densities using spectrophotometric analysis of the reduced MRSA growth and loss of turbidity indicating that efficient lysis happened. Phages isolated from sewage were enriched on MRSA bacterial host in liquid lysate, purified by centrifugation, 0.2 µm filtration, chloroform treatment, and stored as glycerol stocks at −70° for long-term preservation (Bonilla et al., 2016;2016.). The results of the spot test and spectrophotometric assay gave mostly the same results. Twenty-four MRSA isolates lysed by phages possibly due to the lack of prophages in these isolates, and they may be found in the 6 others not lysed by any of the isolated phages. Our results were in concordance with a previous study (Jensen et al., 2015). The titer of phages here in this study was determined using plaque assay to be 1.04 × 10^6^ pfu/ml. Plaques appeared as small-sized pinheads.

Negative staining of purified viruses and electron microscopy is the gold standard in the identification of bacteriophages. This is due to the absence of any highly conserved universal barcoding genes like the *16S rRNA* gene in bacteria and the internal transcribed spacer (ITS) region in fungus (Tan et al., 2020). Morphological characteristics of phages can be used for their classification. While there is a variety of different morphological phage types, most *S. aureus* phages possess icosahedral capsids with double-stranded (ds) DNA as a genome and belong to the C*audovirales* order (tailed phages) (Giuseppe Aprea et al., 2015). TEM results showed that the heads of the isolated phages are icosahedral, tails are long non-contractile, and also they appeared with very short non-contractile tails.

We have selected three known *S. aureus* bacteriophages for further characterization. Phage K and phage P68 were isolated in this study and that was confirmed by PCR amplification of the endolysin gene of phage K and phage P68 minor tail protein gene. This explains the broad range of lytic activity of our isolated staphylophage lysate that displayed 80 % lytic activity of the total strains assayed. This was consistent with the results drawn from previous literature that states that phage K is a well-known staphylophage and displayed 79% lytic activity on the strains assayed (Abatángelo et al., 2017). The polymerase gene of phage 44AHJD could not be detected by PCR, despite its high resemblance to phage P68 (Hrebík et al., 2019, Vybiral et al., 2003). The two identified phages by PCR Phage K and phage P68 follow the family *Myoviridae* and *Podoviridae* respectively under the order *Caudovidales* (tailed phages) which is the most commonly reported candidate for bacteriophage therapy due to its typical lytic infection cycle, a required property in antimicrobial therapy (Adesanya et al., 2020, Mohammed-Ali and Jamalludeen, 2015).

The nature of the isolated phage DNA was confirmed by restriction digestion. NEB cutter and map of the genome of the studied phages were checked for restriction sites for endonucleases. Proteome analysis using SDS-PAGE, about 4 distinct proteins were found in the gel. The protein size ranged between 42 and 140 kDa. A protein band of 65 kDa was reported as a major tail sheath protein based on sequence homology with phage K (Rasool et al., 2016).

We can summarize from the above results that phages with lytic ability specific to human MRSA isolates were successfully isolated from sewage and characterized. Our further aims in the future include next generation sequencing (NGS) and *in vivo* application of these phages in induced animal infection models to make certain of their therapeutic potential.

## 5 Conclusion

It is worth mentioning that this research is the first in Egypt to discuss the isolation of Staphylophages from sewage water with broad host antibacterial potential against MRSA. Using molecular methodologies provide precise identification of phages. Utilizing of lower cost of bacteriophages for clinical use is promising especially in middle-income country like Egypt. Phages can replace antibiotics when they do fail to kill bacteria.

## Funding

This study was funded by the financial support of the national project 33390, Young Research Grant (YRG) 2019, Science and Technology Development Fund (STDF), Ministry of Higher Education and Scientific Research, Giza, Egypt.

## 7 Ethics approval

All samples in the present study were archived and coded instead of patients’ names. The protocol of the study was approved by TBRI institutional review board under Federal Wide Assurance (FWA00010609) and the work has been carried out in accordance with the Code of Ethics of the World Medical Association (Declaration of Helsinki) for Experiments in Humans and its later amendments (GCP guidelines) or comparable ethical standards.

## 8 Availability of data and material

Data and Material are available with the corresponding author upon reasonable request.

## CRediT authorship contribution statement

**Safia Samir:** Conceptualization, Methodology, Resources, Writing – original draft, Writing – review & editing, Supervision, Investigation, Data curation. **Amira El-Far:** Methodology, Validation, Investigation. **Sami Mohamed Nasr:** Resources, Validation, Methodology, Investigation, Writing – review & editing. **Rania Mahdy:** Validation, Investigation. **Fatima Samir:** Methodology, Investigation, Resources, Validation. **Hend Okasha:** Resources, Methodology, Investigation, Writing – review & editing, Data curation.

## Declaration of Competing Interest

The authors declare that they have no known competing financial interests or personal relationships that could have appeared to influence the work reported in this paper.
